# Resource Management for Massive Internet of Things in IEEE 802.11ah WLAN: Potentials, Current Solutions, and Open Challenges

**DOI:** 10.3390/s22239509

**Published:** 2022-12-05

**Authors:** Arshad Farhad, Jae-Young Pyun

**Affiliations:** Wireless and Mobile Communication System Laboratory, Department of Information and Communication Engineering, Chosun University, Gwangju 61452, Republic of Korea

**Keywords:** IEEE 802.11ah, Internet of Things, machine-to-machine (M2M), low-power communication, medium access control layer (MAC)

## Abstract

IEEE 802.11ah, known as Wi-Fi HaLow, is envisioned for long-range and low-power communication. It is sub-1 GHz technology designed for massive Internet of Things (IoT) and machine-to-machine devices. It aims to overcome the IoT challenges, such as providing connectivity to massive power-constrained devices distributed over a large geographical area. To accomplish this objective, IEEE 802.11ah introduces several unique physical and medium access control layer (MAC) features. In recent years, the MAC features of IEEE 802.11ah, including restricted access window, authentication (e.g., centralized and distributed) and association, relay and sectorization, target wake-up time, and traffic indication map, have been intensively investigated from various aspects to improve resource allocation and enhance the network performance in terms of device association time, throughput, delay, and energy consumption. This survey paper presents an in-depth assessment and analysis of these MAC features along with current solutions, their potentials, and key challenges, exposing how to use these novel features to meet the rigorous IoT standards.

## 1. Introduction

Low-power wide area networks (LPWANs) are designed for static and mobile Internet of Things (IoT) applications that demand energy-efficient long-distance communication [1]. LPWAN technologies have been utilized in the IoT domains, including identification, sensing, computation, cloud, and fog-edge computing [2]. IEEE 802.11ah is one of the significant LPWAN technologies, including narrowband-IoT (NB-IoT) and Sigfox, widely used for the IoT [3]. Specifically, the IoT needs a communication protocol that can meet application needs, such as extended range, low energy consumption, low latency, and scalability [4].

Current low-power IoT communication systems are mainly categorized as wireless personal area networks (WPAN) and low-power wide-area networks (LPWAN). A detailed comparison between LPWAN and WPAN technologies is illustrated in Table 1. The WPAN technologies comprise ZigBee and Bluetooth Low Energy (BLE). ZigBee and BLE offer a medium data rate ranging from a few hundred kbps to Mbps at a few meters distance [5,6]. Among WPAN technologies, BLE requires less energy than ZigBee (i.e., IEEE 802.15.4 standard). On the other hand, the LPWAN technologies, including Long-Range (LoRa) [4], SigFox, NB-IoT [7], and IEEE 802.11ah [8] emphasize long-range communication ranging from 1 km to 50 km. The data rate of LPWAN technologies ranges from a few hundred bps to a few Mbps. In the LPWAN technologies, the NB-IoT and SigFox are IoT-specific 5G technologies that operate in licensed frequency channels, while other LPWAN technologies operate in the ISM band [9,10]. In terms of transmission range, SigFox has the highest range of 50 km, followed by LoRaWAN with a range of 20 km in suburban areas, Nb-IoT with 15 km, and IEEE 802.11ah has the lowest range of 1 km. However, because of the limited transmission range of BLE and ZigBee and the insufficient throughput, they are limited to several IoT use cases such as indoor positioning [11,12] and healthcare systems [13]. Consequently, there is a need for a low-power and massive IoT communication system, requiring substantial throughput and a few km of transmission range. To address this gap, the IEEE 802.11ah, commonly known as Wi-Fi HaLow, is introduced as an LPWAN technology since it has the highest data rate and transmission range of all WPAN technologies [8].

IEEE 802.11ah [20] targets IoT and machine-to-machine (M2M) communications [21,22,23] owing to its promising features such as long range and low power. The IEEE 802.11ah is now intended for the smart grid (e.g., smart meters, smart houses, smart utility), smart agriculture, smart industry, extended range, and smart healthcare [24,25,26,27,28]. For instance, the smart grid is intended to facilitate intelligent power transmission and consumption in urban areas via two-way communication between smart meters and the grid system [29]. In a smart grid scenario, a home area network (HAN) can be used for automation, where the smart appliances communicate the consumed energy to the smart meter. The smart meter transmits the energy consumption statistics to an intermediate controller, such as a neighborhood area network (NAN). A single NAN contains a few hundred intelligent meters organized in HANs. Finally, a WAN is designed to connect various NANs and transmit data to the grid. The IEEE 802.15.4 and BLE cannot satisfy the throughput requirement of a high-density network over a long communication range [30]. In contrast, IEEE 802.11ah provides a medium access protocol for a network with massive stations (STAs) connectivity, long-range, and energy constraints [31].

IEEE 802.11ah can support up to 8191 STAs on a sub-1 GHz band within a 1 km range while providing at least 100 kbps throughput [32]. The IEEE 802.11ah supports three types of STAs: traffic indication map (TIM), non-TIM, and unscheduled. TIM STAs essentially adhere to both delivery traffic indication map (DTIM) and TIM beacons and use a traffic-aware power-saving strategy known as “target wake-up time (TWT)”. The AP is responsible for scheduling the sleep and waking times of the communicating STAs using TWT. On the other hand, the non-TIM STAs negotiate with the AP for their subsequent channel-access intervals. Finally, unscheduled STAs may be broadcast whenever the channel is available. Based on an association identifier (AID) of the STAs, the AP is responsible for time slot distribution to STAs in a beacon interval (BI).

Despite the growing relevant literature in the IEEE 802.11ah domain, many important questions remain unanswered.

1. What are the currently deployed use cases of the IEEE 802.11ah in the industry?
2. What are the advantages and disadvantages of the current literature to support IoT applications?
3. What MAC feature of the IEEE 802.11ah WLAN is the most suitable and widely adopted for improving resource efficiency?
4. What impact do deployment choices, such as channel plan and MAC feature, have on the performance of the IEEE 802.11ah WLAN?
5. What kind of traffic (UL/DL) harms the network performance of the IEEE 802.11ah WLAN network?

The primary goal of this survey is to answer these questions and provide insights into the strengths and limits of the legacy IEEE 802.11ah WLAN in the IoT domain.

### 1.1. Related Survey Articles

The specification for Wi-Fi HaLow gives a thorough overview of the PHY and MAC layers [33]. However, the decision making of parameter configuration and optimization (e.g., resource allocation) is left open to developers and academic researchers, allowing them to construct and develop solutions (e.g., prototype or mathematical-based models) for rigorous IoT applications. In recent years, several studies have surveyed the IEEE 802.11ah, its PHY and MAC layers, and solutions designed for low-power IoT applications. Table 2 provides the main focus and brief description of the main topics covered by the existing survey articles on IEEE 802.11ah WLAN.

Mainly, these survey articles focus on PHY and MAC layer techniques with limitations and future directions. Compared to other relevant surveys, as illustrated in Table 2, this survey article presents an in-depth assessment, and critical analysis of these MAC features along with current solutions, highlighting their potentials and key challenges and exposing how to use these novel PHY and MAC features to meet the rigorous IoT application requirements.

### 1.2. Motivation and Contributions

In contrast to the published surveys and tutorials highlighted in Table 2 that present many characteristics or provide a comprehensive evaluation of the IEEE 802.11ah WLAN system, where channel access mechanisms, RAW optimization techniques, PHY, and MAC layers are the major topics, our survey mainly focuses on resource allocation in a MAC layer with its adoption for M2M and massive IoT networks. The contribution of this article, compared to the surveys and tutorials presented in Table 2, is as follows.

1. First, we briefly describe the IEEE 802.11ah, including deployed use cases of the IEEE 802.11ah WLAN and the MAC layer features.
2. Second, the current solutions in the literature are thoroughly studied and assessed from several aspects, along with their shortcomings for different MAC features of the IEEE 802.11ah WLAN.
3. Third, the potentials and challenges of the IEEE 802.11ah are identified, and open research issues and future research directions are briefly discussed.

### 1.3. Structure of Survey

**Figure 1:**
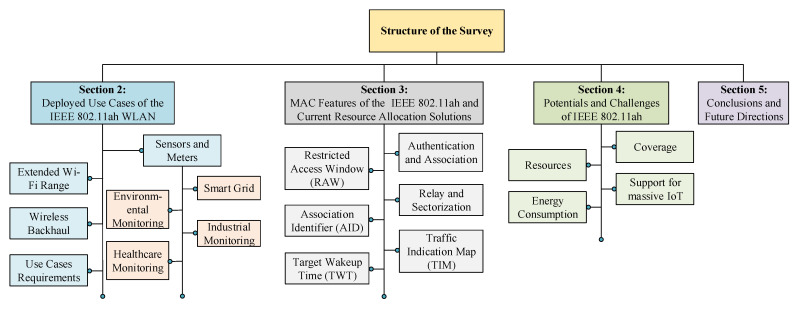
Outline of the survey paper.

Figure 1 presents the detailed structure of the survey paper and provides a section-wise overview. Section 2 presents the IEEE 802.11ah applications, deployed use cases, and their requirements. Section 3 provides an overview of the MAC features of the IEEE 802.11ah and current resource allocation solutions alongside their key objectives and shortcomings. Finally, Section 4 discusses the potentials and challenges of IEEE 802.11ah, whereas Section 5 provides concluding remarks on this survey paper and highlights future directions.

**Table 2:** A brief summary of existing IEEE 802.11ah WLAN surveys and tutorials.

Ref.	Year	Main Focus of Surveys	Brief Description of Main Topics Covered
[31]	2012	advantages and challenges	highlighted the IEEE 802.11ah standardization efforts and explored the benefits and challenges of physical (PHY) and medium access control (MAC) layer methods
[34]	2013	channel access enhancement	aimed to describe the channel access (e.g., MAC layer) improvements made to the IEEE 802.11ah specification to improve machine-type communication (MTC) performance
[35]	2013	overview of PHY and MAC layer	surveyed the MAC layer enhancements, including power-saving features, support for many STAs, efficient medium access methods, and throughput improvements for meeting the expected system requirement
[36]	2014	energy consumption in the MAC layer	explored the energy consumption in the MAC layer along with a performance evaluation of IEEE 802.11ah in four popular M2M situations, namely farm monitoring, smart metering, industrial automation, and animal monitoring
[30]	2015	mechanisms and challenges	in-depth overview of the fundamental mechanisms and the use of these mechanisms in smart cities with related open issues are discussed
[37]	2015	PHY and MAC layer feature	summarized the issues and solutions regarding throughput and network reliability as well as discussed the proposed enhancements to the PHY and MAC layers
[38]	2015	PHY and MAC layer features	provided an overview of the PHY and MAC layers and highlighted how various features might address the issues associated with enabling IoT use cases
[39]	2016	challenges for IoT scenarios	investigated the essential properties of IEEE 802.11ah to satisfy IoT needs and provided a comprehensive assessment of IEEE 802.11ah WLAN
[40]	2017	challenges and future directions	surveyed the state of the art of IEEE 802.11ah WLAN and identified the technical challenges and research directions in this area
[41]	2017	cooperative MAC protocols	offered an in-depth overview of current research on cooperative MAC and relay-based MAC protocols and models desirable categories and problems in the IEEE 802.11ah standard
[42]	2017	study of M2M communications	presented state-of-the-art M2M technologies, focusing on IEEE 802.11ah, and explored the future challenges and envisioned opportunities
[43]	2019	study of LPWAN technologies	explored the LPWA technologies, focusing on technical parameters of IEEE 802.11ah, LoRa, and NB-IoT
[44]	2019	MAC layer features	presented a detailed review of the research on MAC improvement for wireless real-time control applications
[45]	2019	MAC layer features	provided a comparative study of MAC protocols for the IoT was performed to provide insights into IoT applications, considering their characteristics, limitations, and behavior, along with challenges and open research issues
[46]	2021	suitability for IoV and IoT-enabled networks	surveyed IoT communication technologies and highlighted the benefits of the Internet of Vehicles (IoV) and IoT-enabled networks
[47]	2021	machine learning techniques	presented several Wi-Fi applications of machine learning, providing researchers an in-depth review of the major trends, open issues, and future recommendations
[48]	2021	challenges in mobile IoT networks	provided a comprehensive view of the challenges introduced by mobility in IoT networks under different IoT standards related to intelligent transport, smart agriculture, smart cities, and industry that involve collecting measurements and performing coordinated actuation
[8]	2021	PHY and MAC layer features	presented a detailed review of the WiFi Halow, comprising analysis of the PHY and MAC layers, open challenges, and addressed future orientation
[49]	2022	MAC layer features	provided a comprehensive assessment of various MAC protocols and highlighted issues and limitations of the MAC layer

## 2. Deployed Use Cases of the IEEE 802.11ah WLAN

IEEE 802.11ah has three main categories of use cases: network for sensors and meters, extended Wi-Fi range, and wireless backhaul [38,39,40,49]. In this section, applications regarding each use case are introduced.

### 2.1. Sensors and Meters

IEEE 802.11ah is recommended for M2M and IoT applications based on sensors and meters due to its low power consumption, long range, and suitability in a highly dense network. As an example of the IEEE 802.11ah application, the smart grid [50] mainly consists of smart meters, smart appliances, renewable energy resources (e.g., wind turbines and solar panels), and an energy management system. Furthermore, the hierarchical communication network of the smart grid can be categorized as HAN, NAN, and WAN [24,51].

NEWRATEK, one of the Wi-Fi solution providers, designed a chip for IoT devices based on IEEE 802.11ah to support smart grid application [52,53]. The primary aim of the suggested solution is to increase the network lifetime due to longer sleep time. In addition, it provides support for both AP and STA roles.

#### 2.1.1. Smart Grid-Meter to Pole

Wireless sensor networks [54,55] allow customers and utilities to hand over, monitor, and accomplish energy resources efficiently. They are widely adopted by smart meters, where the sensors proactively report the usage (readings) to the AP after a scheduled interval. Thus, utility companies can manage electricity demand and respond efficiently [50]. Smart grid applications include smart metering, home energy management, load management, demand and response, outage detection, renewable generation, distributed storage, distributed generation, vehicle to grid, etc. [24,25,50,51,56,57,58,59,60].

As an example of smart grid application devices, Ref. [61] shows the IEEE 802.11ah-based Wi-Fi module kit, supporting 1/2/4 MHz channel bandwidth for both AP and STA roles.

#### 2.1.2. Environmental/Agricultural Monitoring

This category is the outdoor use case of IEEE 802.11ah in sub-GHz. Applications that come under surveillance include measurement of wind speed, wind direction, humidity, temperature, water level, soil condition, pollution information, plant and crop condition, animal habitat and location, and gathering of disaster information [26,62,63,64,65].

To support agriculture monitoring using IEEE 802.11ah, a remote monitoring system was designed to prevent crop damage caused by wild animals such as boars or deer. The existing remote monitoring and alarming system notifies the farmer and identifies the trapped wild animals [66].

#### 2.1.3. Industrial Monitoring

IEEE 802.11ah recommends use cases for monitoring and controlling equipment and manufacturing processes. Industrial monitoring primarily aims to provide quality products and safety measurements. Thousands of sensors are required in the automation process at input/output points, where processes such as refining petroleum and manufacturing iron and steel can be monitored to increase productivity and quality and ensure reliability [67].

**Figure 2:**
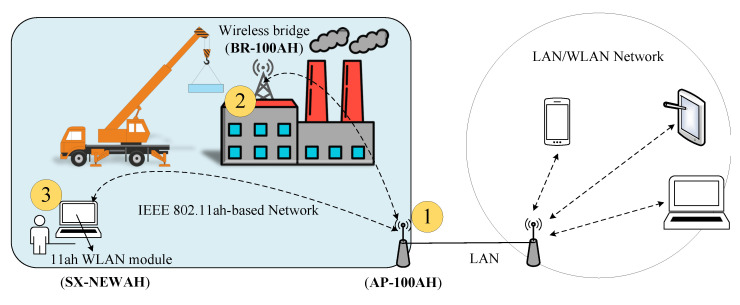
Use case of IEEE 802.11ah wireless bridge BR-100AH in industrial application [68]: (1) AP-100AH: IEEE 802.11ah-compatible AP; (2) BR-100AH: IEEE 802.11ah-compatible wireless bridge; and (3) SX-NEWAH: IEEE 802.11ah-compatible WLAN module [69].

For industrial monitoring, Silex Technology has proposed a solution, Wireless Bridge (BR-100AH), based on IEEE 802.11ah [68]. BR-100AH supports communications at sub-1 GHz and provides a much longer range than the case of 2.4 GHz/5 GHz, more device connections per AP, radio interference avoidance with 2.4 GHz/5 GHz radios, and much better radio coverage due to better wall penetration and diffraction around obstacles. Simple deployment for industrial monitoring of BR-100AH is shown in Figure 2, where a wireless bridge connects a non-wireless device (10BASE-T/100BASE-TX network device) to IEEE 802.11ah WLAN. With sub-GHz radio, various non-wireless devices can easily be connected to a wide-range IEEE 802.11ah WLAN. Furthermore, the BR-100AH employs WPA3 Wi-Fi authentication to ensure safe and secure use of wireless communication at an office, factory, etc., where higher security is required.

#### 2.1.4. Healthcare Monitoring

IEEE 802.11ah can be utilized for indoor healthcare monitoring systems, including hospitals/clinics, elderly care, and personal fitness monitoring [70]. For example, in the case of a hospital/clinic, a patient can be monitored by deploying different sensors that can monitor heart rate, blood pressure, oxygen in the blood (SOPO2), temperature, electrocardiogram, etc. [71]. The caregivers use the information collected from the patient to provide the required medication.

Elderly care is an essential part of health monitoring. Sensors are deployed on the patient to monitor urgent notifications, including fall detection and pill bottle monitoring [72]. Many health problems that affect the elderly are caused by falls, which are the leading cause of accidental deaths. A fall detection system sends an alert signal to the caregiver to provide timely medical assistance [73]. Further, to monitor the daily medicine intake, a smart pill bottle provides an easy way to send sensor data directly to the user’s mobile device [74]. In addition, personal fitness includes weight and heart rate monitoring, the data collected over a certain period. This information is analyzed periodically to help the users control, maintain, and improve their lifestyles.

### 2.2. Extended Wi-Fi Range

A primary scenario of IEEE 802.11ah is the extended range of hotspots offering Internet access over WLAN. Wi-Fi is regularly used for hotspot applications globally. These domains can benefit from the extended area by using lower frequency bands in IEEE 802.11ah. The distinctive frameworks of a long-range hotspot include homes, campuses, and shopping malls [75]. Besides hotspots, IEEE 802.11ah is also considered a leading choice for traffic offloading, a solution to mobile traffic explosion. Due to the massive coverage of IEEE 802.11ah, it can be applied to mobile traffic offloading in outdoor environments [76].

### 2.3. Wireless Backhaul

**Figure 3:**
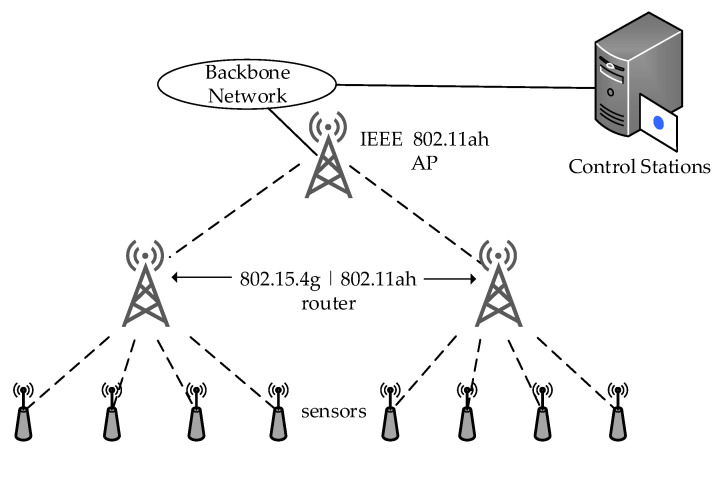
Wireless backhaul scenario of IEEE 802.11ah.

Figure 3 shows a backhaul aggregation of sensors having battery power restrictions induced by IEEE 802.15.4g. It is primarily designed for low-rate wireless personal area networks and is a premier choice for low-power, long-range STAs due to its applicability to intelligent utility networks [6]. In addition, IEEE 802.11ah provides an appropriate feature of a backhaul link to receive the aggregated data of leaf STAs. As a result, the data can be conveyed to an IEEE 802.11ah AP for scrutiny and retrieved from the Internet. Moreover, IEEE 802.11ah performs these tasks without the scarcity of network throughput and consistency, even if co-existing with IEEE 802.15.4g, due to the S1G feature [39,77,78].

### 2.4. IEEE 802.11ah Use Cases Requirements

IEEE 802.11ah supports IoT and M2M applications and scenarios. Therefore, based on its promising features, such as long-range and low power, it supports low data rate applications (e.g., up to 100kbps) within the range of 1 km [79]. Furthermore, another requirement for the IEEE 802.11ah is that it supports more than two thousand STAs in outdoor environments [33]. IoT and M2M STAs are usually battery-powered and are expected to operate in low-power mode to conserve energy. Therefore, IEEE 802.11ah must provide enhanced power-saving techniques for battery-powered operation with a long lifetime [80]. Furthermore, Table 3 summarizes the basic requirements for IEEE 802.11ah WLAN use cases, where the IEEE 802.11ah-enabled devices can transmit data either continuously, periodically, or in a burst. The devices continuously transmit data to the server in the continuous data transmission mode. Whereas the periodic data transmission is performed by the devices for a certain amount of time and then goes into an energy-saving mode (i.e., sleep mode). Finally, burst data transmission refers to a period when the data from a device is sent at irregular intervals.

## 3. MAC Features of the IEEE 802.11ah and Current Resource Allocation Solutions

**Figure 4:**
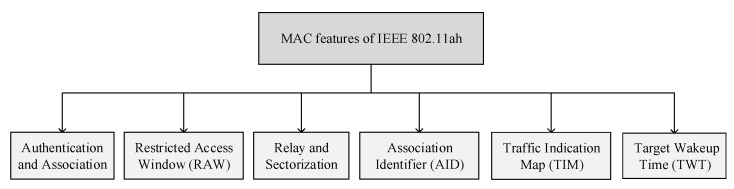
Categorization of the MAC layer features of the IEEE 802.11ah WLAN.

This section briefly discusses the promising MAC layer features of IEEE 802.11ah, including authentication and association, restricted access window (RAW), relay and sectorization, association identifier (AID), traffic indication map (TIM), and target wake-up time, as shown in Figure 4. In this section, first, we thoroughly discuss the abovementioned MAC layer features of the IEEE 802.11ah WLAN, their potentials, and issues. Second, we discuss the current resource allocation solutions for each MAC layer feature. Finally, the traffic type, performance evaluation, shortcomings, and simulator tool utilized in each solution are highlighted.

### 3.1. Authentication and Association

**Figure 5:**
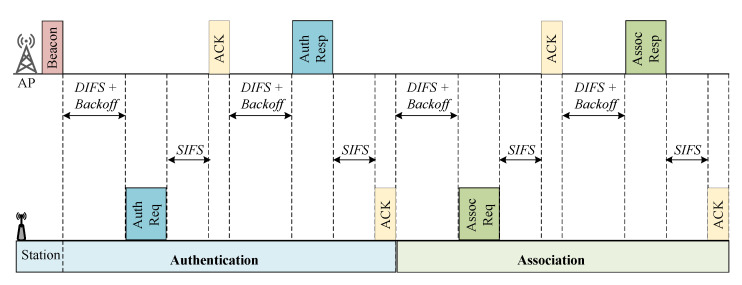
The station link setup process with access point in IEEE 802.11ah [86].

When communication to an AP is required, all STAs before the communication should initiate authentication/association setup with the AP, as indicated in Figure 5 [81]. An STA transmits an authentication request (AuthReq) and association request (AssocReq) to an AP, allowing the AP to discover the existence and capabilities of the STA [22,82,83,84]. By returning the authentication response (AuthResp) and association response (AssocResp) to the STA, the AP assigns AID. Unfortunately, STAs utilize the DCF channel access method during the link setup phase, causing a collision between authentication and association messages [84,85]. Due to the collisions between authentication and association messages, link setup between STAs and AP might be time-consuming when several STAs attempts to associate simultaneously with the AP. This becomes problematic at the IEEE 802.11ah with the massive STAs deployed for IoT applications.

The IEEE 802.11ah suggests two techniques for overcoming this issue: (1) centralized authentication control (CAC) and (2) distributed authentication control (DAC).

1. **Centralized Authentication Control:** The AP modifies the proportion of STAs permitted to transmit AuthReq messages in the CAC method. The AP creates a threshold and broadcasts it through beacon frames to all STAs. The beacon frame comprises network information that the AP regularly delivers to advertise the network existence and synchronize all STAs. When an STA starts authentication, it randomly generates a value within the [0, 1022] range. If the beacon delivered from the AP has a random value smaller than the threshold at the STA (i.e., available range of threshold values: [0, 1022]), the STA tries to transmit the AuthReq to the AP. If not, the authentication/association procedure is delayed until the next beacon arrives. The AP should dynamically adjust the threshold to limit the number of STAs that may access the channel in a single BI and guarantee that all STAs can associate with the AP rapidly.
2. **Distributed Authentication Control**: The DAC technique divides a BI into authentication control slots (ACSs). STAs randomly choose a BI and ACS to communicate their AuthReq to the AP. If STA fails to authenticate, it retransmits the AuthReq in the next $m^{th}$ BI and $i^{th}$ ACS, where *m* and *i* are calculated using the truncated binary exponential backoff approach [8].

#### Related Studies Concerning Authentication/Association

Related studies concerning authentication/association are presented here, and their specific details are highlighted in Table 4. The primary purpose of these existing schemes is to reduce the authentication/association time of STAs with AP.

The authors in [87] proposed an analytical model to resolve the collisions of authentication and association messages. First, the study showed the impact of the AuthReqs parameters in a single ACS that affect the network performance in average authentication time. Then, they found the number of successful AuthReqs with a function of the number of STAs that have chosen the BI. Finally, the average authentication time for the chosen STA is computed. Their proposed method showed that the association parameters were set up efficiently, and the link setup time was close to the minimum.

Similarly, the authors in [88] studied the handshakes in the IEEE 802.11ah for improving the association time of STAs using an analytical model. In particular, they focused on the CAC, a default method utilized by the IEEE 802.11ah. They developed analytical and heuristic methods to manage CAC parameters and improve their efficiency adaptively. Their results show that their proposed solutions decrease the link setup time during the association of STAs with AP.

A performance evaluation of the association time was studied in [89], where STAs are randomly deployed in a 400 m range around an AP. This study focused on the performance evaluation of the association time, implementation, and validation of IEEE 802.11ah RAW in ns-3 [90]. Similarly, the authors of [84] examined the authentication and association procedures of the IEEE 802.11ah. Their study revealed that the association procedure might take several minutes and argued that a new method is required to prevent collision between AuthReqs and traffic from already-associated STAs with AP. In addition, [81] demonstrated that the optimal group of STAs can result in minimum association time. However, for the link setup procedure, the assumptions presented in [81] are the constant probability of successful transmission and the AP intelligence of how many STAs will be asking for connections, which are unrealistic.

**Table 4:** Existing solutions on authentication and association along with shortcomings.

Ref.	Year	Traffic Type	PerformanceEvaluation	Shortcomings	Evaluation Tool
[84]	2015	UL	association time	does not solve the issues of coexistence data and association frames	ns-2
[88]	2015	UL/DL	link setup time	collision is still an issue owing to large association requests and hidden STAs	ns-2
[89]	2016	UL/DL	association time and throughput	no solution was proposed to reduce the association time	ns-3
[87]	2016	UL/DL	throughput	link setup time increases linearly with the increasing number of STAs since it does not entirely resolve the collision issue owing to a large association requests	analytical
[87]	2016	UL/DL	throughput	collision is still an issue owing to large association requests	analytical
[81]	2017	UL/DL	association time	during the link setup process, the AP knows the number of STAs in advance, which is unrealistic	analytical
[91]	2018	UL/DL	association time	association and PS-Poll frames create overhead, which can cause excessive energy consumption	ns-3
[92]	2019	UL/DL	association time	the existing study does not determine the best data rate for massive IoT devices registration that can speed up the registration process	ns-3
[82]	2020	UL/DL	association and authentication	their proposed FASUS technique does not guarantee fairness if the user modifies the MAC address for fast association with the AP	ns-3
[83]	2020	UL/DL	delay and energy consumption	the proposed fast key re-authentication (FKR) method does not consider the traffic requirements	MATLAB
[93]	2021	UL	association delay	their proposed method does not consider the existence of both association and data exchange frames	ns-2
[94]	2022	UL/DL	association time	it does not consider the heterogeneous traffic	analytical

To effectively register STAs with an AP, the slotted-CSMA/CA with time division multiple access (TDMA) was proposed in [91]. In their proposed method, contention-based slotted-CSMA/CA enables STAs to transmit the AuthReq through randomly chosen backoff slots. Their simulation findings showed improved contention-free transmission compared with the traditional IEEE 802.11ah with CSMA/CA.

In addition, a simulation study was presented in [92] to evaluate the association time of STAs. Their study using multi-rate capabilities during association showed improved performance by 5–15% relative to the association at a fixed transmission data rate.

Recently, a new association mechanism named fast association based on speculating the number of STAs (FASUS) was proposed in [82]. This method aimed to improve the authentication and association process of STAs. To minimize unnecessary retransmission, the FASUS approach retransmits the AssocReq/AssocResp pair depending on retry counts rather than timers. In addition, the number of STAs inside the network is used to adjust the threshold dynamically (i.e., available range of threshold: [0, 1022]).

The fast key re-authentication (FKR) method aimed to speed up the re-authentication process by using the nonce (i.e., a pseudo-random number generated as part of an authentication procedure to guarantee those past communications cannot be reused in replay attacks) of a single STA to introduce unpredictability into the system [83]. Owing to the single nonce introduced in each authentication round, their proposed FKR protocol may reduce the authentication procedure to just two messages. The detailed evaluation of the FKR method has shown that it is more effective in terms of time delay and energy usage.

To address the association issue, the authors proposed automating the cluster head selection using a low-energy adaptive clustering hierarchy (LEACH) protocol in [93]. Their simulation results revealed that their LEACH-based proposed method using the cluster of STAs efficiently reduces the association delay.

Authors [94] proposed an analytical model for examining the association process under jamming to assess the interference effect. Their findings indicated that the jammer might fail the association process and prevent network access, rendering IoT applications. In addition to the association process, they also proposed countermeasures for various jamming attempts to defend against the problem.

### 3.2. Restricted Access Window (RAW)

**Figure 6:**
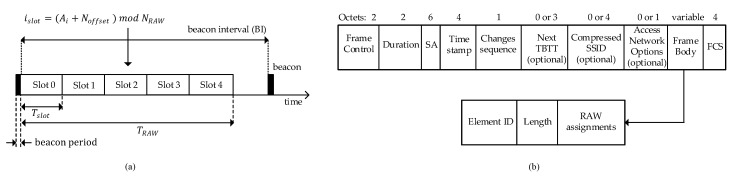
Time allocation in BI: (**a**) RAW of IEEE 802.11ah; (**b**) beacon frame and RPS element.

A RAW aims to save power in STAs and provide high-channel-utilization services to several STAs participating in the communication. In IEEE 802.11ah, a beacon interval (BI) is composed of a RAW, which is divided into time series called “slots”, as shown in Figure 6a. After every BI, thousands of STAs attempt to access the medium for communication in the BI. In each BI, a RAW period is divided into a series of time slots having equal duration. However, the number of slots assigned in each RAW period can differ based on the data transmission requests [95]. An AP is responsible for assigning slots to a group of STAs during $N_{RAW}$, as shown in Figure 6a. The remaining STAs not involved in the transmission go into sleep mode to save energy.

However, RAW-related information is included in the RAW parameter set (RPS) element that is contained within the frame body subfield of the beacon frame, as shown in Figure 6b [20]. In the RPS element, each RAW assignment subfield contains the necessary parameters to restrict the medium access to one or more STAs within a RAW. The RPS element further defines RAW start time, RAW duration $N_{RAW}$, RAW group, AIDs, and eligible STAs to contend for their assigned time slots. Furthermore, a parameter called cross-slot boundary is included in the beacon, which defines the behavior of the RAW. An STA can cross the slot boundary of its assigned slots for data transmission. If the cross-slot boundary is not permitted, STAs can only access the channel if the remaining time in the assigned slot boundary is adequate to complete the data communication. Otherwise, the STAs are not allowed to initiate transmission.

Based on the information including $N_{RAW}$ and time slot period $T_{slot}$ contained in RPS, individual STAs compute the number of slots to be assigned. In each RAW, these slots are indexed from 0 to ($N_{RAW}-1$), where $N_{RAW}$ indicates the total number of slots. Each STA decides its slot index $i_{slot}$, which shows the permitted slot for accessing the medium based on the slot assignment function:

(1) $$\begin{array}{c} i_{slot}=\left(A_{i}+N_{offset}\right)\;\mathrm{mod}\;N_{RAW}, \end{array}$$

where $A_{i}$ is the position index of an AID of the STA, and $N_{offset}$ is an offset value provided to address the fairness among the STAs indicated in the TIM. $N_{offset}$ is determined via an offset field in the beacon frame from an AP and may change throughout one or more BIs so that the slots assigned to the STAs vary. Moreover, the AP prevents the constant end-to-end delay by changing the $N_{offset}$ value in the beacon [50].

#### Related Studies Concerning RAW Optimization

Related studies concerning RAW optimization are presented here, and details are shown in Table 5. The current solutions for RAW optimization in the literature primarily focus on enhancing the network performance in terms of throughput [96,97,98,99,100,101,102,103,104,105,106,107,108,109,110,111,112,113,114,115,115], channel efficiency/utilization [97,104,116,117,118], and energy consumption [59,102,105,111,112,119,120,121,122,123,124].

A traffic-adaptive RAW optimization algorithm (TAROA) was proposed to dynamically adapt the RAW parameters considering the underlying traffic conditions [100]. The TAROA scheme is triggered each time a target beacon transmission time (TBTT) is sent. The TAROA scheme calculates the packet transmission interval of each STA based only on packet transmission information gathered during the previous BI by the AP. Based on this projected transmission frequency, the TAROA estimates the RAW characteristics and allocates STA to RAW slots. Their simulation findings indicate that, compared to EDCA/DCF, the TAROA method may significantly increase the throughput performance of IEEE 802.11ah.

The channel access slot (CAS)-based channel access protocol [96] aims to divide the RAW period into slots (downlink and uplink accesses). More concretely, the proposed scheme adjusts the number CASs and their length based on the number of STAs contending in RAW. In each CAS, a selected group of STAs compete to transmit data. To ensure fair access to all STAs, a periodic reset of back-off is assumed in combination with the random choice of the CAS performed by the STAs. As a result, in the CAS protocol, STAs remain in the sleeping mode for more than 98% of the time, consequently increasing the network lifetime. Moreover, a higher value of CAS slots can lower the probability of collision and, thus, reduce channel contention and increase the packet delivery ratio. However, the CAS algorithm does not consider the collision that has occurred in the previous BI since an STA can randomly select the same slot for transmission, which results in a high possibility of collision. Therefore, if a higher value of CAS is chosen for the lower number of STAs, the channel utilization is possibly reduced.

To boost the network performance, the authors in [98] dynamically modified the RAW period dependent on the number of STAs. First, STA cannot cross its allotted slot boundary when transmitting data in the RAW. Second, the STA can cross the slot boundary; however, this STA is not allowed to transmit data in another slot, which is already allocated to another STA. Consequently, their proposed method may significantly improve channel efficiency by restricting the amount of STAs during this contention phase. Thus, it enhances the normalized throughput gains by up to 210% and 770% in a network of 128 and 256 groups, respectively. Furthermore, the collision is possibly reduced during the handover process of slots between multiple groups. However, several mini-slots within a RAW may remain unused, resulting in under-utilization of the channel during the process mentioned above.

A new MAC enhancement algorithm proposed in [97] aims to simultaneously adjust the RAW period by considering the relation between the expected number of STAs and the duration of RAW to increase the channel efficiency. Based on the success probability of STAs, the ideal size for uplink and downlink in the RAW period is determined. The analysis shows that this scheme outperforms a typical IEEE 802.11ah in terms of success probability, network throughput, and channel utilization due to the dynamic nature of the RAW period. However, the application content is restricted to one type of traffic; therefore, it does not apply to massive IoT applications due to their heterogeneous traffic nature. Traffic load is essential in determining the optimal size of a slot in the RAW uplink access. Without considering the traffic load of STAs, an optimal size of RAW cannot be guaranteed in a massive IoT-based network.

The renewal access protocol (RAP) [116] has two strategies for undertaking collisions capably and attaining high network throughput performance in a congested network: strategies without grouping (RAP) and with grouping (G-RAP). RAP dynamically allocates several slots to individual groups by considering the back-off transmission attempts, whereas G-RAP aims to enhance channel efficiency by considering transmission attempts. As a result, it efficiently solves the channel contention and achieves acceptable throughput performance regardless of the group size. Moreover, G-RAP achieves better fairness due to assigning the same number of transmission attempts to each group. However, G-RAP can cause a significant delay due to higher back-off time during high contention.

A hybrid MAC for energy efficiency based on the contention reservation scheme (CRS) is presented in [122]. This scheme aims to enhance the network lifetime based on the number of STAs per group. The proposed CRS consists of three main phases: grouping, negotiation, and RAW slot assignment. During the first phase, the AP forms disjoint groups of STAs, and in the second phase, STAs with a low energy budget can start negotiating with the AP based on the TWT procedure before going into sleep mode. Finally, during the third phase, the STAs begin transmitting the PS-Poll messages for slot reservation, and then the AP schedules the RAW slots and transmits the number and duration of the slots in the subsequent BIs. Meanwhile, the STAs contend for the channel in the assigned time slots for buffered packet transmission. However, a mathematical CRS model enhances the network lifetime for a successful uplink data communication by up to 55% overall. This is because the AP assigns a slot to only those STAs that have successfully transmitted the PS-Poll messages during contention. Therefore, it results in contention-free access and reduces energy consumption. Nevertheless, the larger data packets compared to that of a PS-Poll frame in a large network consisting of 1500 STAs causes a higher contention delay of 62 s during transmission.

The sequential transmission scheme (STS) [104] was proposed to reduce channel contention. In STS, an AP is responsible for allocating RAWs to each group based on the number of transmitting STAs and the total traffic generated by each group. The AP computes the network load in the UL direction and resources required by each group by considering the duration of PS-Poll frames, whereas the DL traffic is known to the AP. As a result, STS achieves better channel utilization because STAs access the channel based on their allocated time; hence, no slots are wasted due to collisions or idle channels during data communication in a RAW. Similarly, STS accomplishes high network throughput, i.e., 2.4 and 2.2 Mbps, under Modulation and Coding Scheme (MCS7 (6.5 Mbps) conditions and MCS5 (5.2 Mbps), respectively. However, while transmitting data at MCS3 (2.6 Mbps), STS suffers longer delays and lower throughput due to the high contention and lower transmission rate.

Reference [100] aimed to adjust the RAW parameters dynamically based on the current network traffic load by considering the UL transmission to enhance the balance between channel contention and utilization. Their proposed method, known as TAROA, is mainly based on two steps: (1) transmission interval estimation and (2) RAW slot assignment. During the transmission interval estimation phase, TAROA computes the packet interval of each STA based only on the packet transmission information obtained during the last BI. The second step calculates the RAW parameters and assigns STAs to RAW slots based on the expected transmission frequency. In TAROA, each STA is expected to transmit packets predictably. The simulation results show that under the dynamic number of STAs (i.e., 1536), high traffic load (i.e., T = 1.275 Mbps), and Poisson rate of 0.1 s, the TAROA algorithm achieves 0.83 Mbps throughput, as compared to EDCA/DCF, which achieves 0.63 Mbps throughput. However, TAROA significantly suffers under the Poisson rate of 5 s and achieves 0.077 Mbps throughput out of T = 0.1425 in the “without hidden nodes” case compared to EDCA/DCF, which achieves 0.09712 Mbps throughput. This is because TAROA fails to adapt due to the highly topological change and, thus, reduces the network throughput.

A dynamic energy-aware window control algorithm is described in [102], which mainly aims to enhance energy efficiency and network throughput by precisely adjusting the RAW duration based on the number of STAs per group. The method assumes a single-hop star topology for smart grid-dense network communication. The method is based on the gradient descent approach, an efficient technique for less working load and repository capacity to achieve maximum energy efficiency. The simulation results demonstrate that the proposed RAW method achieves up to 50% improved results for packet delivery ratio because the algorithm adjusts the RAW size based on the group scale compared to the legacy RAW mechanism. Likewise, the proposed algorithm shows a 20% improvement in terms of energy consumption when compared to the existing RAW. However, the proposed method fails to sustain its performance due to the high probability of collision, leading to excessive energy consumption, low throughput, and long delay as the number of STAs increases per group.

The RAW size is generally adapted dynamically by accounting for the group size to increase the network throughput. First, reference [101] suggests that the groups are organized based on size. Second, the RAW is further divided into two independent sections, called “subframes,” while the length of each subframe is adapted based on the group size. The proposed scheme enhances the throughput as the larger duration of the RAW period is allocated to a relatively smaller number of STAs to avoid high contention. However, the proposed scheme can result in the underutilization of the channel since the larger size of the RAW period is assigned to a group containing fewer STAs.

Energy-aware window control schemes are presented by proposing a retransmission mechanism to enhance energy consumption [59,123]. The schemes reduce the retransmission attempts by adopting the slots in a RAW based on the number of active STAs. However, in most cases, the AP assigns the same slot to many STAs, since the AP is unaware of whether the STAs are in active or sleep mode. The scheme presented in [123] divides the number of STAs into groups, where an AP computes the number of active STAs in each group based on the transmission attempts, and accordingly, the slots are computed in a RAW. An STA contends to gain access to a slot in the RAW period randomly, and if it fails to access the slot, it tries to access the following slot in the same RAW. The proposed scheme shows an enhanced network lifetime of up to 12.3% as compared to the existing RAW mechanism. Correspondingly, the packet delivery ratio is improved, in general, up to 7.5%, due to the re-use factor of empty slots in a RAW period.

Reference [117] proposed a traffic-aware grouping technique to achieve fairness. Their proposed method computes the expected channel utilization of each group based on their traffic requirements, then determines the contention probability. Their study was validated through ns-3, which improved the channel utilization by 5.5% and 9.7% in random grouping and simple utilization, respectively. In addition, their proposed method improved channel fairness by avoiding starvation and uneven distribution of channel resources among distinct groups.

A TDMA and CSMA/CA-based channel access mechanism is proposed for IEEE 802.11ah to improve the throughput performance by considering the traffic load [109]. In CL-MAC, the STAs are initially classified based on their Traffic Profiles (TPs). TPs represent the features, such as the payload of STAs and priority. TPs are further divided into real-time or non-real-time traffic patterns. Based on the traffic priority, TDMA slots are assigned to the STAs based on the group density. The CL-MAC also proposes Guaranteed Time Slots (GTSs) for high-priority traffic. GTSs are only assigned based on the requests received by an AP from a designated STA. The remaining STAs can use the CSMA period for those without access to the TDMA period. The CL-MAC algorithm shows better performance when compared to the EDCA mechanism of IEEE 802.11ah.

The influence of mobility models, Random Walk, Gauss–Markov, and Random Waypoint, was analyzed in the IEEE 802.11ah network by varying the traffic and network density [112]. Their simulation results revealed that the Random Waypoint produced better results in throughput. However, in a high network density case, the performance is degraded by increasing the number of STAs due to high contention. Recently, the authors in [115] proposed a grouping method by splitting the RAW into two sub-RAWs. Their proposed RAW method allows the STAs to group in a similar size inside each sub-RAW.

### 3.3. Association Identifier (AID)

**Figure 7:**
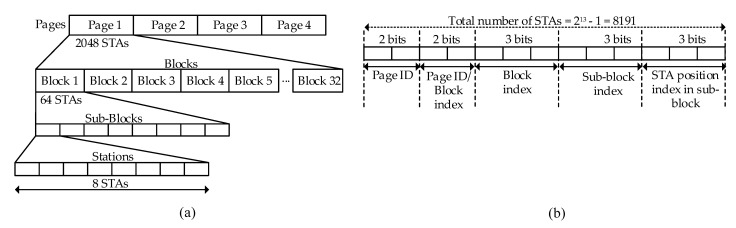
Association identifier (AID): (**a**) hierarchical structure of AID; (**b**) AID structure for STA.

An AP is responsible for assigning slots to individual STAs and a group of STAs during the time for which the allowed STAs can contend for the channel. An AID is unique in a network, containing 13 bits, divided into 4 levels: page, block, sub-block, and STA’s index in sub-block. A page is divided into 32 blocks of equal length and 8 sub-blocks each. An individual block and sub-block can support up to 64 and 8 STAs. Similarly, a sub-block comprises eight bits, corresponding to an STA position index. This hierarchical construction enables IEEE 802.11ah to support more than 8000 STAs, as shown in Figure 7 [125], and can be adapted to facilitate the grouping of STAs much more quickly. The STAs with different priorities and traffic patterns can be easily grouped to utilize the resources efficiently. Hence, grouping can be used to save power, allocate resources, and for the efficient channel, access [35].

During the network deployment, the AP allocates AIDs to STAs based on (2), which illustrates how an AID ($A_{i}$) is computed and how a slot is assigned to a specific STA during a communication [125].

(2) $$\begin{array}{c} A_{i}=PageID\times 4+\left(\frac{PageID}{BlockIndexExtention}-1\right)\\ \times 8+(BlockIndex-1)\times 8+(SubBlockIndex-1)\\ \times 8+(BitPositionOfStation), \end{array}$$

where PageID is the page identifier and $\frac{PageID}{BlockIndexExtention}$ consists of two bits; BlockIndex, SubBlockIndex, and BitPositionOfStation comprise three bits. Equation (2) shows the calculation of a unique number per STA based on the hierarchical structure illustrated in Figure 7b [125].

#### Related Studies Concerning AID, Overhead, and Backoff

The current solutions regarding AID, overhead, and backoff mechanism in the IEEE 802.11ah WLAN are presented here, and their shortcoming and other features are shown in Table 6.

The collision chain mitigation (CCM) algorithm presented in [126] mitigates serious collisions in IEEE 802.11ah dense networks (e.g., smart grid) by using the simultaneous allocation of slots in the RAW duration based on hidden STAs. To mitigate the collisions, CCM follows four steps: (1) detection, (2) interruption, (3) mediation, and (4) identification. First, to detect a collision, an AP checks abnormally long duration by examining the energy block in a channel and considering it as an existence of a collision chain. Second, after collision detection, CCM interrupts the transmission and broadcasts a notification signal. Third, during the phase of mediation, those STAs can continue transmission who have already attempted but failed. Last, in the identification step, the AP collects the transmission start time of STAs to identify the hidden nodes based on the overlapping transmission among different STAs in each group and updates the carrier sensitivity. As a result, the CCM algorithm achieves a lower delay of up to 5 ms compared to the conventional mechanism, which reaches around 28 ms. Furthermore, CCM attains considerably low collision by limiting the number of hidden STAs during group formation. Furthermore, CCM achieves higher throughput by 139% and 146% with six and eight groups, respectively, by limiting the number of hidden STAs to less than 1% in each group. However, the CCM performance degrades (up to 17% less) in a relatively small network of 400 STAs compared to the conventional case in terms of throughput.

A power-saving scheduling technique was developed in [127]. The primary aim was to schedule smart meters to uplink/downlink traffic to reduce the network overhead. Their proposed method also considers the dynamic allocation of the AID to STAs communicating in the network based on their average service time, allowing STAs with comparable service characteristics to occupy subsequent AIDs. Their proposed method showed improved results regarding overheads, throughput, average wake-up time, and energy consumption. Another similar method was presented in [128] to reduce the encoded bitmap length within the same hierarchical structure.

**Table 6:** Current solutions on association identifier (AID), overhead, and backoff in IEEE 802.11ah WLAN with shortcomings.

Ref.	Year	Traffic Type	Performance Evaluation	Shortcomings	Evaluation Tool
[128]	2014 2015	UL	bitmap reduction	their proposed method can cause excessive overhead owing to the association and authentication frames, PS-Poll, and ACK etc., still challenging	unknown
[129]	2016	UL/DL	throughput, delay, association time	limiting contention and RAW size calculation over dynamic network	ns-3
[126]	2016	UL/DL	collision mitigation	collisions remain due to longer sleep duration and number of hidden STAs remain in each group during the regrouping process	MATLAB
[127]	2017	UL/DL	throughput, energy, overhead	it does not take into account energy efficiency as the similarity index	MATLAB
[127]	2017	UL/DL	overhead, energy	it completely ignores energy consumption and unnecessary wake-up of STAs and the support for RAW schemes	MATLAB
[130]	2018	UL	delay and retransmission	optimizing contention window size in a small IoT network does not help in reducing the number of collisions in massive IoT scenario	OPNET
[131]	2018	UL/DL	throughput, delivery ratio, delay	it does not take into heterogeneous traffic requirements while optimizing the contention window size	ns-3
[132]	2018	UL/DL	fairness, delay, interference	EDCCA and Q-learning backoff diminish the packet delivery rate of IEEE 802.11ah because they limit STAs’ channel access opportunities	analytical
[133]	2018	UL/DL	scalability, throughput, latency, energy	their proposed method does not consider the STAs status (i.e., either asleep or awake) is still challenging	ns-3
[134]	2019	UL/DL	throughput, latency	it is not compatible with the current RAW of the IEEE 802.11ah WLAN	analytical
[135]	2021	UL/DL	throughput, packet collision and loss rate	their proposed method does not consider re-grouping for load balancing to improve throughput	ns-3

A distributed MAC protocol supporting a large number in IEEE 802.11ah was proposed for multi-hop communication in [129]. Simulation findings indicated that the suggested protocol enhances throughput, network coverage range, and average end-to-end packet latency compared to 802.11ah. However, most STAs operate in sleep mode in IEEE 802.11ah WLAN, and it is projected that the network would have a considerably greater collision probability at the beginning of a BI than at the end. To mitigate these limitations, the authors in [130] proposed an adaptive contention window technique in which the contention window size is optimized at the beginning of the BI and subsequently decreased after the transmission is successfully completed. Through simulations, it was demonstrated that the suggested method might improve IEEE 802.11ah WLAN performance. Similarly, the collision problem was mitigated with another backoff mechanism in [131], where the authors have developed an analytical model to optimize the contention window size. Their analytical and simulation results showed improved throughput, delivery ratio, and delay performance.

The authors in [132] proposed a learning-based self-coexistence control method for IEEE 802.11ah to decrease interference impact on IEEE 802.15.4g. First, the authors utilized the energy detection clear channel assessment method (EDCCA) on IEEE 802.11ah-enabled STAs to sense the ongoing data communication of IEEE 802.15.4. Second, the authors proposed a Q-learning method by utilizing the backoff mechanism of the IEEE 802.11ah to avoid interference caused by IEEE 802.15.4 packets. Their simulation results showed improved spectrum-sharing performance among both IEEE 802.15.4 and IEEE 802.15.4g.

A simulation study was conducted in [133] to examine the concurrent use and performance of TIM and RAW on scalability. In the presence of DL traffic, the RAW mechanism restricts UL performance by delaying it until the next available channel access opportunity and giving DL priority. As a result, UL-only networks have different RAW performance than networks with bidirectional or DL traffic.

To prevent collisions, an analytical model in [134] was designed to employ association IDs to determine a deterministic backoff value. Existing techniques are compared to the deterministic backoff technique’s performance. According to the findings, their proposed method yields a greater throughput and lower latency than previous deterministic backoff systems.

### 3.4. Relay and Group Sectorization

#### 3.4.1. Relay

To extend the transmission range between a root AP and STAs, IEEE 802.11ah suggests the use of a relay to serve IoT applications demanding high coverage. Relays consist logically of a relay AP and a relay STA. Relay APs are associated with STAs, while relay STAs are linked to root APs. The root AP sends packets to the relay STA for DL transmission. The relay STA then forwards the sent packets to the relay AP. To simplify the forwarding approach, the relay connection between an STA and the root AP is limited to two hops.

#### 3.4.2. Group Sectorization

Group sectorization utilizes time-division multiplexing with space-division multiplexing [30,136]. It splits a BSS coverage area into sub-areas (i.e., geographical regions), with each sub-area including a subset of STAs, to alleviate hidden terminal issues, traffic congestion, and interference. The sectorization is accomplished by the AP sending or receiving through several antenna beams spanning multiple BSS sectors [49].

#### 3.4.3. Related Studies Concerning Relay and Group Sectorization

Table 7 provides an in-depth overview of the various resource allocation MAC protocols suggested for relay and group sectorization. The highlighted approaches in Table 7 support relay [108,129,137,138,139,140], and group sectorization [141] is supported in IEEE 802.11ah. These approaches can increase the network coverage and capacity. These approaches are compared concerning their unique objectives and their shortcomings highlighted. Relay and group sectorization approaches enhance scalability and improve coverage but lack energy efficiency and cannot function in a heterogeneous scenario.

Among these approaches, reference [137] proposed a hierarchical MAC framework for relay to extend the range. Their proposed method improved the throughput by mitigating interference and lowering energy consumption. Similarly, the authors in [129] presented a distributed MAC protocol focusing on multi-hop communication to support many IoT STAs. Their simulation findings demonstrated that their proposed protocol outperforms IEEE 802.11ah in terms of performance, network coverage range, and average end-to-end packet latency. Reference [129] was improved by the authors in [108] using estimating the RAW size based on traffic loads and providing relay node assistance for STAs using various data rates. The relay nodes distributed bandwidth dynamically to STAs belonging to various relay groups. Another study presented a relay-based IEEE 802.11ah WLAN based on a cross-layer approach to optimize the energy consumption of the STAs [138]. A simulation study through an analytical model was conducted in [139] to evaluate the effectiveness and achievable range with dual-hop relay, as suggested by IEEE 802.11ah.

A sector-wise STA grouping system for fast channel access was proposed in [141]. First, the coverage area is divided into unique sectors by the AP. Second, each sector is further divided into various groups based on the number of STAs, and each group within a sector is then allotted particular RAW slots. Finally, STAs inside each group in different sectors use a spatial orthogonal access method to access the allocated RAW slots. Compared to the standard DCF and IEEE 802.11ah grouping methods, their simulation findings demonstrated that their proposed method might significantly increase network performance while concurrently minimizing the delay.

### 3.5. Traffic Indication Map (TIM) Segmentation

**Figure 8:**
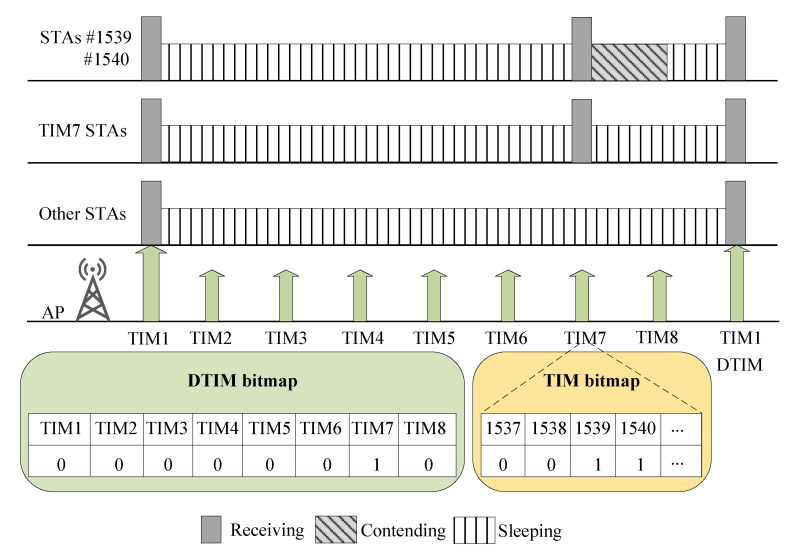
Example of TIM beacon with the non-cross and cross slot boundaries of RAW mechanism [133].

A TIM element, known as TIM beacon, is used inside each beacon frame by the AP to indicate a list of STAs with buffered data packets [98]. In the case of no buffered packets, the STA stays asleep. Otherwise, a PS-Poll frame is transmitted to recover the buffered data packets. However, the AP transmitting DL traffic inside a beacon frame for some STAs causes a bottleneck. IEEE 802.11ah addresses this issue with an enhanced power-saving mechanism known as TIM segmentation. This method splits the TIM information into *N* segments (e.g., TIM groups). The TIM beacon carries information for each TIM group. The Delivery Traffic Indication Map (DTIM) beacons are used for TIM group-level signaling, while TIM beacons are employed at the STA level. Periodically, all STAs wake up at a designated time to receive the DTIM signal. All STAs in the TIM group check if the AP has data waiting for their TIM group. In the case AP has data waiting for STAs, they wake up and listen to the TIM beacon. Otherwise, all STAs go back to sleep mode. As shown in Figure 8, the AP has only pending data for TIM Group 7. Consequently, STAs in TIM Group 7 wake up and listen to the corresponding TIM signal, while other stations return to sleep until the subsequent DTIM announcement. In addition, when the beacon for TIM Group 7 is received by the STAs, signaling that the AP has pending data for STAs 1539 and 1540, the remaining STAs of TIM Group 7 continue to sleep while STAs 1539 and 1540 compete for channel access to obtain the data from the AP.

#### Related Studies Concerning TIM Segmentation

Table 8 provides an overview of the various related studies suggested for TIM segmentation. Some existing studies have examined the TIM segmentation performance from different perspectives, including the energy consumption for DL traffic [142,143,144,145]. In contrast, other related studies are interested in using TIM segmentation and RAW to support both UL and DL [111,133,146,147,148], to provide and support heterogeneous services.

The authors in [143] examined the performance of the TIM segmentation for the DL traffic under different network settings. The findings indicated a trade-off between delay and energy consumption; shorter DTIM intervals may reduce delay, but it can increase energy consumption.

To solve the TIM segmentation problem, [144,145] presented a solution that dynamically modifies the STA membership. Their method issue a primary and secondary AID to an STA. As a result, the STA becomes a member of two distinct TIM groups, enabling it to transition between the groups and reorganize its traffic to optimize total resting time without delaying data transmission.

Reference [146] proposed an analytical model to reduce energy consumption. The model comprised a RAW group composed of one DL TIM and one UL segment. Based on a set of closed-form equations, the model estimated the average energy used by an STA and anticipated its battery lifespan.

A simple and precise mathematical model [147] was adopted to manage a protected interval during which only a subset of low-power STAs with sporadic traffic transmit their PS-Polls to retrieve DL packets indicated by TIM elements to reduce energy consumption for low-power STAs and increase throughput for the powered STAs with saturated traffic.

To improve the efficiency of the heterogeneous network (e.g., with different requirements of the IoT applications), the authors proposed an analytical model using a joint TIM segmentation with RAW [147]. In addition, the authors studied the impact of the throughput and average energy consumption. They improved the throughput and energy consumption compared to the legacy RAW mechanism.

### 3.6. Target Wakeup Time (TWT) for Energy Saving

TWT reduces the energy consumption of STAs with intermittent data transmission. TWT enables STAs to decide when and how often they wake up to receive data from the AP or send data to the AP. According to the TWT mechanism, the TWT service period (SP) is the amount of time a sleeping STA is awake and able to receive or send packets. Therefore, the STAs do not need to wake up unnecessarily to receive beacons from the AP. As a result, the STA can stay in low-powering saving mode (i.e., sleep mode) for a long time.

#### Related Studies Concerning TWT and Energy Saving

Table 9 provides an in-depth overview of the various resource allocation MAC protocols suggested for TWT. Some earlier works have focused on TWT [149,150,151,152]. As RAW may be used to safeguard TWT STAs from collisions with other STAs, Ref. [149] suggested interleaving TWT STAs. As a result, their TWT may be covered by a single RAW, lowering RAW indication overhead and transmission delay.

The article in [150] focused on how RAW and TWT may help in the reduction of energy consumption. First, the authors introduced an analytical model that determines the typical energy use across a RAW session. Next, the IEEE 802.11ah simulator that was modified for this purpose with an energy life-cycle model for RAW and TWT was used to compare these findings. Next, the authors utilized RAW to analyze energy use in various scenarios. Finally, a TWT was used to assess energy use. Their findings demonstrate that the analytical model has a maximum divergence from simulations of 10% when the capture effect (CE) is present and 7% when it is not. Furthermore, when there is more traffic, RAW consistently outperforms CSMA/CA, and using additional slots has been shown to have superior energy efficiency, up to 76%, and dramatically increasing latency.

Authors [151] investigated the energy consumption of RAW and TWT STAs coexisting. Their findings showed that the presence of RAW STAs might have a detrimental impact on the energy efficiency of TWT STAs. However, a suitable channel access scheduling can offset this effect without compromising the throughput performance of RAW STAs. Clock drift is one of the issues with TWT since it causes devices to stop closely adhering to the schedule, therefore missing the planned transmission time, which increases the active time and energy consumption. Recently, the authors in [153] proposed a PRAW with a unique analytical model for minimizing the channel timeshare, average latency, and energy consumption. The important aspect of their analytical model is the analysis of short PRAW slots, which reduces computing complexity and achieves high precision.

**Table 9:** Current solution concerning TWT with shortcomings.

Ref	Year	Traffic Type	Performance Evaluation	Shortcomings	Evaluation Tool
[89]	2016	UL	throughput, packet loss rate, latency	TIM segmentation is not taken into account	ns-3
[149]	2018	UL	throughput	no solution is proposed for RAW mechanism optimization	analytical
[152]	2018	DL/UL	packet delivery rate, energy consumption	RAW optimization is not taken account	ns-3
[150]	2019	DL/UL	latency	the transmission time should be know to utilize TWT	ns-3
[151]	2019	DL/UL	energy consumption	clock drift might cause missing the planned transmission time, which may increase the active time and, consequently, energy consumption	ns-3
[153]	2021	UL/DL	throughput, delay, energy consumption	a tradeoff exists between delay and energy consumption, influenced by the traffic intensity	analytical and ns-3

## 4. Potentials of IEEE 802.11ah and Challenges

This section highlights the potentials and challenges of IEEE 802.11ah in the massive IoT, M2M, and Heterogeneous Internet of Medical Things (Het-IoMT) applications. The requirements can be differentiated to envision the challenges within the IoT, Het-IoMT, and M2M communications. For example, massive STAs transmitting traffic (i.e., homogeneous or heterogeneous) simultaneously or periodically, features such as low energy consumption, network coverage, and a long sleep interval. The rest of this section presents an overview of the features used by the IEEE 802.11ah to tackle the challenges faced by IoT applications.

### 4.1. Network Coverage

Network coverage refers to the area in which wireless signals are transmitted. IEEE 802.11ah support thousands of STAs on a sub-1 GHz band within a 1 km coverage area. However, some of the applications need a higher range than 1 km due to the nature of the operation (e.g., smart grid [52,53]). The network coverage of WLANs in an urban environment is highly impacted by two primary sources, namely, multipath and external WLAN interference [37]. The adverse effects of multipath are dominant if omnidirectional antennae are used. However, multipath effects can lead to higher end-to-end delay and channel attenuation, leading to intersymbol interference (ISI) in WLANs.

IEEE 802.11ah fulfills the coverage requirement by presenting a 1 MHz channel bandwidth with a new feature of the MCS index, MCS10. In addition, it also provides support for 2, 4, 8, and 16 MHz. Unfortunately, extending the transmission range requires a larger symbol duration than the legacy IEEE 802.11 due to the support of such narrow bandwidths. However, due to longer symbols and guard time intervals, IEEE 802.11ah is robust to ISI in outdoor and indoor environments [37].

Furthermore, due to the support of MIMO, IEEE 802.11ah benefits from spatial diversity. This feature improves the received signal quality of the IEEE 802.11ah link; hence, longer links are possible. In addition, IEEE 802.11ah provides an appropriate feature of a backhaul link to accommodate the aggregated data and helps increase coverage. Furthermore, the IEEE 802.11ah specification considers multi-hop features with relay nodes to further extend the coverage [39].

### 4.2. Resources

Currently, some technologies operate in the most widely used frequency band of 2.4 GHz (IEEE 802.11, IEEE 802.15, BLE, etc.). As a result, these technologies suffer from interference, degrading the network performance concerning packet loss, high energy consumption, and long network delays. Moreover, due to massive IoT device connectivity, long-range, and diverse resources, the 2.4 GHz band does not look suitable for IEEE 802.11ah. On the contrary, communication problems, such as inter and intra-interference, which is very destructive in CSMA-based channel access techniques, are aggravated. Nevertheless, the IEEE 802.11ah standard is envisioned to operate below 1 GHz resulting in better coverage and less interference. This feature of the IEEE 802.11ah seems attractive for the IoT, M2M, and Het-IoMT applications, where thousands of devices are likely to coexist. In addition, to accommodate massive IoT devices, IEEE 802.11ah recommends using RAW to provide high-channel-utilization services to several STAs/groups of STAs participating in the communication [154].

### 4.3. Support for Massive IoT

IoT, M2M, and Het-IoMT-based networks consist of thousands of STAs. However, the network suffers high collision because STAs try to associate with the AP simultaneously, thus leading to a severe collision. To limit channel contention, IEEE 802.11ah offers a promising feature called an authentication control mechanism (ACM), which allows only a small group of STAs for association in a BI. In addition, during the process of simultaneous communication, collisions occur frequently. These excessive collisions result in reduced network throughput, increased end-to-end communication latency, and excessive energy consumption during the retransmission process; thus, finding an appropriate technique to reduce the collision is challenging in the field of IoT. Furthermore, when multi-APs with large-scale associated STAs are considered in a massive IoT network, APs are presumed unsynchronized, and no coordination between them is used. Under this scenario, high interference between the APs is expected, and a notable decay in throughput might occur.

Previous standards of IEEE 802.11 support only up to 2007 STAs by a single AP due to the limited number of AID that can be assigned during the association/authentication [30,33]. On the contrary, IEEE 802.11ah uses a novel AID structure, as shown in Figure 7, to support 8191 STAs by a single AP. IEEE 802.11ah groups the thousands of STAs based on similar characteristics by efficiently utilizing the AID structure, proving support for massive IoT STAs.

### 4.4. Energy Consumption

The IoT devices are driven by a battery and should work for months or years based on the application requirements. Hence, low power consumption becomes a crucial aspect of increasing the battery life of IoT, M2M, and Het-IoMT STAs. To prolong the network lifetime, IEEE 802.11ah uses the TWT mechanism [152]. Based on TWT, AP schedule the wake-up time of STAs and their channel access attempts. The wake-up information between an AP and an STA is exchanged utilizing an association request and association response frames. Thus, the STAs with a low energy budget can start negotiating with the AP based on the TWT procedure before going into sleep mode.

Similarly, IEEE 802.11ah uses a shorter header, implicit acknowledgment, and speed frame exchange (i.e., Transmission Opportunity), which helps in extending the battery lifetime of STAs by shortening the transmission time and keeping them awake for shorter periods.

## 5. Conclusions and Future Directions

IEEE 802.11ah, known as Wi-Fi HaLow, is a viable next-generation Wi-Fi technology envisioned for long-range, low-power, and large-scale IoT applications. This survey offers a detailed review and analysis of the current developments in IEEE 802.11ah WLAN. First, this survey presented a detailed summary of the current surveys and tutorials and their targeted areas, use cases of IEEE 802.11ah with deployed scenarios, and a description of IEEE 802.11ah WLAN along with MAC features. Second, the current solutions for different MAC features of the IEEE 802.11ah WLAN were thoroughly studied and assessed from several aspects, along with their shortcomings. Thirdly, the potential of the IEEE 802.11ah and its challenges were discussed.

In conclusion, IEEE 802.11ah combines the benefits of Wi-Fi and low-power communication technologies, supports a high data rate, and can meet the performance requirements of various IoT applications with tailored approaches such as large-scale networks, low power, and QoS. In a massive IoT network, UL traffic is dominant. Therefore, the RAW mechanism can be adapted to minimize collisions by modifying appropriate parameters. These parameters include RAW group length, the number of slots in a RAW, and the number of STAs. As a result, the IEEE 802.11ah WLAN performance in terms of throughput, energy consumption, and latency could be increased. In addition, TIM segmentation may be used to reduce energy consumption in low-powered STA and DL traffic. In a massive IoT network, a mix of RAW, TIM segmentation, TWT, and other characteristics may be deployed concurrently to fulfill heterogeneous traffic demands or QoS requirements. Moreover, massive access communication based on machine learning is a new trend in IoT wireless communication. With resource-constrained device features, intelligent resource allocation, signal processing, channel estimate, and transceiver design may increase IEEE 802.11ah WLAN performance.

Finally, in the future, we anticipate that Wi-Fi HaLow will play a significant role in massive IoT networks and be extensively adopted for IoT when more off-the-shelf solutions become available.

## Figures and Tables

**Table 1:** A comparison between IEEE 802.11ah and current wireless technologies for IoT.

	LPWAN				WPAN	
**Features**	**IEEE 802.11ah**	**LoRaWAN**	**NB-IoT**	**Sigfox**	**BLE**	**ZigBee**
Spectrum	Sub GHz	868 MHz (ISM) in EU	Sub GHz	Sub GHz	2.4 GHz (ISM)	2.4 GHz (ISM)
Bandwidth	1–16 MHz	125/250/500 KHz	180 KHz	100 KHz	2 MHz	5 MHz
Modulation	OFDM	chirp spread spectrum	QPSK	BPSK	GFSK	OQPSK
Topology	star	star	cellular	star	star	mesh
Data rate	150 Kbps	300–500 Kbps	250 bps	100 bps	1–2 Mbps	10–250 Kbps
Range	1000 m	20 km	15 km	50 km	30 m	100 m
Power consumption	Low	Low	High	Low	Low	Low
Mobility support	Yes	Yes	No	No	Yes	Yes
Localization	Yes	Yes (TDoA)	No	Yes (RSSI)	Yes	Yes
Bidirectional	Yes	Yes	Yes	Limited	Yes	Yes
Advantages	high data rate	long distance	long distance	long distance	medium data rate	short distance
Shortcomings	medium distance	low data rate	low data rate	low data rate	short distance	low data rate
**References**	[8]	[4,12,14]	[7,15,16]	[9,17]	[5,18]	[6,19]

**Table 3:** Summary of IEEE 802.11ah-based applications under sensors and meters, extended Wi-Fi range and wireless backhaul use cases, and their requirements [49].

Applications	Ref.	Traffic Type	Latency	Throughput	Network Lifetime
Smart grid	Section 2.1.1[50,61]	continuous, periodic, and burst	Low	High	Months to Years
Environmental and agricultural monitoring	Section 2.1.2[62,63]	periodic and event-based	High	Medium	Years
Industrial/Building monitoring	Section 2.1.3[68,69]	periodic and burst	Low	High	Years
Health monitoring	Section 2.1.4[70,71]	periodic and event-based	Low	High	Years
Extended Wi-Fi	Section 2.2[75,76]	burst	Low	High	Years
Wireless backhaul	Section 2.3[39]	periodic and burst	Low	High	Years

**Table 5:** Current solutions on RAW optimization of the IEEE 802.11ah WLAN with shortcomings.

Ref.	Year	Traffic Type	Performance Evaluation	Shortcomings	Evaluation Tool
[96]	2014 2015	UL	throughput	typical DCF design reduces performance for a network with a high number of STAs	MATLAB
[97]	2014	UL	channel efficiency	it examines the active STAs, even though traffic load and energy consumption are equally crucial factors in IoT	analytical
[98]	2014	UL	throughput	it does not optimize the number of groups and RAW duration	analytical
[121]	2015	UL	energy, delay	it completely ignores the unnecessary wake-up of STAs	analytical
[99]	2015	UL	throughput	the improvement is suitable only for traffic of the same kind	analytical
[116]	2015	UL	channel efficiency with success probability	channel contention still an issue in a heterogeneous IoT network with priority-based channel access	analytical
[59]	2015	UL	energy	traffic heterogeneity is not taken into account	MATLAB
[119]	2016	UL	energy	RAW needs to be adjusted dynamically for event detection system	analytical
[120]	2016	UL	energy	traffic heterogeneity in terms of low and high with energy requirements are not considered	analytical
[102]	2017	UL	energy	heterogeneous traffic requirements are not considered	MATLAB
[122]	2017	UL	delay, energy	the DL traffic and the mathematical model is not validated through simulation analysis	analytical
[100]	2017	UL/DL	throughput	it does not always produce better latency performance	ns-3
[104]	2017	UL	channel contention, throughput, delay	the channel resources are not efficiently allocated into different RAW groups	analytical
[101]	2017	UL	throughput	it does not take into account the network traffic and channel contention	analytical
[103]	2017	UL/DL	throughput	priority scheduling creates a RAW group, and fairness concerns that are not considered	ns-3
[102]	2017	UL	energy, packet delivery ratio	RAW size is adjusted based on STAs per group, which does not consider heterogeneous STAs for RAW adaptation	MATLAB
[124]	2017	UL	energy and delay	network conditions are not taken into account for RAW adjustment and grouping of STAs	MATLAB
[100]	2017	UL/DL	throughput, energy	the RAW adjustment is made using just the information available at AP; RAW grouping is inefficient for long-term performance and only considers STAs with identical characteristics	ns-3
[123]	2017	UL	energy, packet delivery ratio	collision in a massive IoT scenario can cause retransmission, leading to channel under utilization	MATLAB
[104]	2017	UL	throughput	dynamic and heterogeneous traffic are not taken into account	OPNET
[105]	2017	UL/DL	throughput, latency, energy	RAW grouping is inefficient for long-term performance since it only considers STAs with identical characteristics	ns-3
[106]	2018	UL/DL	throughput	it failed to consider grouping of STAs in RAW and fairness problems	ns-3
[107]	2018	UL/DL	delay, throughput	heterogeneous STAs (i.e., different MCSs utilized by STAs during communication) within a RAW group are not considered	ns-3
[108]	2018	UL/DL	throughput, delay	saturated level of the network is taken into account always	ns-3
[109]	2018	UL/DL	throughput	their proposed method only classifies the traffic and does not consider RAW adaptation for each classified traffic	ns-3
[110]	2018	UL	throughput, energy	can increase the energy consumption due to retransmission in the UL direction	analytical
[112]	2018	UL/DL	throughput, energy	only analysis of different traffic pattern schemes are presented	ns-3
[111]	2019	UL/DL	delay, energy, throughput	their proposed method does not take into account the dynamic environment of the IoT scenario	ns-3
[117]	2019	UL/DL	channel utilization	channel contention in a massive IoT scenario may cause a collision and leads to the channel under utilization	ns-3
[113]	2019	UL/DL	throughput	traffic-aware STAs partitioning with heterogeneous traffic environment is not considered by their proposed method	analytical
[118]	2019	UL/DL	packet received ratio	predicting the RAW size does not take into account the energy depletion, the dynamic adjustment of RAW, and multi-hop scenarios	unknown
[114]	2020	UL/DL	throughput	the prediction accuracy of the right number of slots in a RAW, contained in BI is still a concern	analytical
[115]	2022	UL/DL	throughput	multi-class with traffic-aware STAs and heterogeneous traffic requirements are not considered	MATLAB

**Table 7:** Current solutions concerning relay and group sectorization in the IEEE 802.11ah WLAN with shortcomings.

Ref.	Year	Traffic Type	Performance Evaluation	Shortcomings	Evaluation Tool
[137]	2015	DL	throughput, energy	it does not deal with problems, such as STA dynamic and traffic heterogeneity	ns-3
[129]	2016	UL/DL	coverage range, delay	limiting contention and calculating RAW size across a dynamic network	analytical
[108]	2018	UL/DL	delay, energy consumption	their proposed method does not provide data aggregation and RAW size prediction	ns-3
[138]	2015	DL	throughput	optimal bandwidth allocation for heterogeneous traffic is not considered in their proposed work	ns-3
[139]	2018	UL/DL	RSSI	their proposed method does not consider the relay location	analytical
[141]	2018	UL/DL	throughput	their proposed method doe not comprehensively demonstrate the simultaneous transmission using multiple channels in different sectors	MATLAB
[140]	2021	UL/DL	throughput, delay	it does not consider delay priority	ns-3

**Table 8:** Current solutions related to TIM Segmentation of the IEEE 802.11ah WLAN with shortcomings.

Ref.	Year	Traffic Type	Performance Evaluation	Shortcomings	Evaluation Tool
[142]	2015	DL	TIM compression	three-level hierarchical TIM compression coding framework can increase the system complexity and energy consumption	unknown
[143]	2016	DL	delay, energy consumption	shorter DTIM intervals may reduce delay at the expense of increased energy consumption	analytical
[144]	2017	DL	energy efficiency	it can increase network delay in terms of traffic delivery in a massive IoT scenario	MATLAB
[144]	2017	DL	bitmap compression	their proposed method does not consider communication delay of the STAs in UL direction and energy consumption	MATLAB
[145]	2018	DL	energy efficiency, quality of service	resource allocation in heterogeneous network is still challenging	analytical
[146]	2018	DL/UL	energy consumption	impact of the RAW is neglected	MATLAB
[147]	2017	DL/UL	throughput, energy consumption	does not consider delay requirements for low-powered STAs	analytical
[133]	2018	DL/UL	scalability, throughput, latency, and energy efficiency	RAW with a large number of slots could increase the delay of the STA in high dense network	ns-3
[111]	2019	DL/UL	throughput, energy, delay	reduction in the BI can cause an increase in the energy consumption of monitoring application and might reduce the overall throughput of the network	ns-3
[148]	2021	DL/UL	collision rate	it does not consider load balancing in slots of a RAW	ns-3

## Data Availability

Not applicable.

## Abbreviations

The following abbreviations are used in this manuscript:

ACM	Authentication Control Mechanism
ACS	Authentication Control Slot
AID	Association Identifier
AssocReq	Association Request
AuthReq	Authentication Request
b/s	bits per second
BI	Beacon Interval
BLE	Bluetooth Low Energy
BSS	Sasic Service Set
CAC	Centralized Authentication Control
CAS	Channel Access Slot
CCM	Collision Chain Mitigation
CRS	Contention Reservation Scheme
DAC	Distributed Authentication Control
DTIM	Delivery Traffic Indication Map
EDCCA	Energy Detection Clear Channel Assessment Method
FASUS	Fast Association Based on Speculating the Number of Stations
FILS	Fast Initial Link Setup
FKR	Fast Key Re-authentication
G-RAP	Renewal Access Protocol with Grouping
GS-DCF	Group-synchronized Distributed Coordination Function
GTS	Guaranteed Time Slot
HAN	Home Area Network
IoT	Internet of Things
IoV	Internet of Vehicles
kbps	kilobits per second
LEACH	Low-Energy Adaptive Clustering Hierarchy
LoRa	Long Range
LPWAN	Low-Power Wide Area Networks
M2M	Machine-to-Machine
MAC	Medium Access Control
MIMO	Multi Input Multi Output
MTC	Machine-Type Communication
NAN	Neighborhood Area Network
NB-IoT	Narrowband-IoT
OFDM	Orthogonal Frequency Division Multiplexing
PHY	Physical Layer
PS	Power Saving
PSM	Power Saving Mechanism
PSMP	Power Save Multi-Poll
RAP	Renewal Access Protocol
RAW	Restricted Access Window
RPS	RAW Parameter Set
RSSI	Received Signal Strength Indication
SP	Service Period
STA	Station
STS	Sequential Transmission Scheme
TAROA	Traffic-Adaptive RAW Optimization Algorithm
TBTT	Target Beacon Transmission Time
TDMA	Time Division Multiple Access
TDoA	Time Difference of Arrival
TIM	Traffic Indication Map
TP	Traffic Profile
TWT	Target Wakeup Time
UAV	Unmanned Aerial Vehicle
WAN	Wide Area Network
WPAN	Wireless Personal Area Network
